# Thermoresponsive Sol–Gel System Incorporating Oleuropein-Rich Olive Leaf Extract for Enhanced Wound Healing and Antibiofilm Activity

**DOI:** 10.3390/gels12040307

**Published:** 2026-04-03

**Authors:** Levent Alparslan, Samet Özdemir, Burak Karacan, Gülşah Torkay, Ayca Bal-Öztürk, Ömer Faruk Tutar, Ece Özcan-Bülbül, Semra Şardaş, Zübeyde Merve Kala, Yıldız Özalp

**Affiliations:** 1 Department of Pharmaceutical Technology, Faculty of Pharmacy, İstinye University, Istanbul 34010, Türkiye; yildiz.ozalp@istinye.edu.tr; 2 Department of Pharmaceutical Technology, Faculty of Pharmacy, İstanbul Health and Technology University, Istanbul 34445, Türkiye; samet.ozdemir@istun.edu.tr; 3 Department of Pharmaceutical Microbiology, Faculty of Pharmacy, İstinye University, Istanbul 34010, Türkiye; burak.karacan@istinye.edu.tr; 4 Stem Cell and Tissue Engineering Application and Research Center (ISUKOK), İstinye University, Istanbul 34010, Türkiye; gulsah.torkay@istinye.edu.tr (G.T.); aozturk@istinye.edu.tr (A.B.-Ö.); 5 Department of Stem Cell and Tissue Engineering, Institute of Graduate Education, İstinye University, Istanbul 34010, Türkiye; 6 Department of Analytical Chemistry, Faculty of Pharmacy, İstinye University, Istanbul 34010, Türkiye; 7 Department of Pharmacy Services, Vocational School of Health Care Services, İstinye University, Istanbul 34010, Türkiye; omer.tutar@istinye.edu.tr; 8 Department of Radiopharmacy, Faculty of Pharmacy, University of Health Sciences, Istanbul 34668, Türkiye; ece.ozcanbulbul@sbu.edu.tr; 9 Department of Pharmaceutical Toxicology, Faculty of Pharmacy, İstinye University, Istanbul 34010, Türkiye; semra.sardas@istinye.edu.tr (S.Ş.); zubeyde.kala@istinye.edu.tr (Z.M.K.)

**Keywords:** oleuropein, sol–gel system, antibiofilm activity, wound healing, topical delivery

## Abstract

Oleuropein, the principal secoiridoid phenolic compound of olive leaves (*Olea europaea* L.), is recognized for its broad-spectrum antimicrobial, antibiofilm, antioxidant, and tissue-regenerative properties. However, its effective local therapeutic application remains challenging due to rapid clearance from the site of administration and limited residence time. In this study, an oleuropein-rich aqueous olive leaf extract was incorporated into a thermoresponsive sol–gel delivery system designed for localized application. The formulation was engineered to remain in a low-viscosity sol state at room temperature and to undergo a temperature-triggered sol-to-gel transition near physiological temperature (~33 °C), enabling in situ gel formation. Oleuropein content was quantified using a validated HPLC method, and the formulation was characterized with respect to physicochemical parameters, thermoreversible gelation behavior, particle size distribution, mechanical properties, and spreadability. Biological performance was evaluated through in vitro cytocompatibility (MTT assay), fibroblast migration (scratch assay), and collagen deposition (Sirius Red staining) in L929 fibroblasts, as well as antibiofilm activity against representative Gram-positive and Gram-negative bacterial strains. The developed sol–gel system demonstrated stable physicochemical characteristics, rapid and reversible thermogelation, suitable mechanical and spreading properties, concentration-dependent inhibition of biofilm formation, and acceptable cytocompatibility within the tested concentration range. Notably, the formulation supported fibroblast viability and collagen-associated responses at optimized concentrations. Overall, the results indicate that the proposed thermoresponsive sol–gel formulation represents a promising strategy for the localized delivery of oleuropein-rich olive leaf extract, combining physicochemical stability with dual wound-healing and antibiofilm functionality.

## 1. Introduction

Olive leaf (*Olea europaea* L.) has attracted growing scientific and industrial attention as a natural source of high-value bioactive compounds. Although historically regarded as an agricultural by-product, contemporary studies have demonstrated that olive leaves contain a complex and pharmacologically relevant phenolic composition, particularly enriched in secoiridoid derivatives. Among these constituents, oleuropein is recognized as the most abundant and biologically active phenolic compound, contributing substantially to the therapeutic potential of olive leaf extracts [1,2].

The qualitative and quantitative distribution of olive leaf phenolics is strongly influenced by geographical origin, environmental stressors, and harvesting conditions. Olive leaves collected from the Aegean region of Türkiye, including the Gömeç area, are exposed to elevated solar radiation, seasonal temperature fluctuations, and moderate water stress—factors known to stimulate secondary metabolite biosynthesis in olive trees. Agronomic and phytochemical investigations have reported that olive leaves harvested from Mediterranean microclimates may exhibit increased oleuropein accumulation as part of the plant’s adaptive defense mechanisms [3,4,5]. Such regional variability is increasingly acknowledged as a critical parameter for the standardization, reproducibility, and quality control of plant-derived pharmaceutical raw materials.

Oleuropein is a highly polar secoiridoid glycoside predominantly localized within the vacuolar compartments of olive leaf cells. Owing to its hydrophilic nature, aqueous extraction represents an effective and selective approach for enriching oleuropein while preserving molecular stability. In contrast to organic solvent-based techniques, water extraction aligns with green chemistry principles and minimizes regulatory and toxicological concerns, particularly for formulations intended for localized biological applications. Several comparative studies have confirmed that aqueous olive leaf extracts can yield oleuropein-rich fractions suitable for pharmaceutical development without compromising biological activity [6,7,8].

From a pharmacological perspective, oleuropein exhibits a broad spectrum of biological effects, including antimicrobial, antibiofilm, antioxidant, anti-inflammatory, and tissue-regenerative activities. Increasing attention has been directed toward its antimicrobial mechanisms, which extend beyond simple growth inhibition. Oleuropein has been shown to interfere with microbial adhesion, alter membrane permeability, and modulate quorum sensing pathways associated with biofilm maturation [9,10,11]. These properties are particularly relevant in biofilm-dominated environments, where microbial communities are embedded within a self-produced extracellular polymeric substance (EPS) matrix that confers structural stability and increased tolerance to antimicrobial agents [12,13,14,15].

Despite these promising biological properties, the successful translation of natural bioactive compounds into effective localized delivery systems remains limited. Both oral and wound environments present physiological challenges, including fluid turnover, enzymatic activity, and mechanical stress, which contribute to the rapid clearance and reduced residence time of applied agents. Consequently, even biologically potent compounds such as oleuropein may exhibit diminished efficacy when administered without an appropriate formulation strategy. This limitation highlights the need for advanced delivery formulations capable of enhancing local retention and controlled release [16,17].

Thermoresponsive (thermoreversible) sol–gel systems have emerged as promising candidates for localized drug delivery due to their ability to undergo reversible phase transitions in response to temperature variations. These systems are typically administered in a low-viscosity sol state and subsequently transform into a semi-solid gel at physiological or near-physiological temperatures. Such behavior facilitates ease of administration while significantly improving residence time at the application site. Recent pharmaceutical studies have emphasized the potential of thermoresponsive sol–gel formulations for localized and mucosal applications, particularly when combined with natural bioactive compounds [18,19,20].

In parallel, increasing emphasis has been placed on evaluating the biological response to locally applied biomaterials. Oleuropein has been associated with modulation of angiogenic pathways and tissue repair processes, suggesting that oleuropein-containing formulations may provide multifunctional benefits beyond antimicrobial action. However, these effects require careful assessment alongside cytocompatibility and toxicological evaluations to ensure biological safety [21,22,23].

The alkaline comet assay, also known as single-cell gel electrophoresis, was used to detect the genotoxic potential of the sol–gel formulation containing olive leaf extract. The alkaline comet assay is widely employed in genetic toxicology as a highly sensitive technique for detecting DNA strand breaks, alkali-labile sites, and DNA adducts, thereby enabling the assessment of genotoxic potential [24].

Based on these considerations, the present study aims to develop a thermoresponsive sol–gel formulation incorporating an oleuropein-rich aqueous olive leaf extract obtained from the Gömeç region and to evaluate its pharmaceutical and biological performance. The study integrates HPLC-based oleuropein characterization, physicochemical and thermoresponsive analysis, mechanical evaluation, and in vitro biological assessment, including cytocompatibility, wound-healing-associated responses, and antibiofilm activity. This approach provides a comprehensive framework for exploring olive leaf-derived oleuropein as a multifunctional natural raw material for localized therapeutic applications.

## 2. Results

### 2.1. Optimized Sol–Gel Formulation

Following systematic formulation development and optimization studies, a thermoresponsive oleuropein-enriched sol–gel system was selected for further characterization. The optimization process involved the evaluation of gelation behavior, viscosity, spreadability, and physicochemical stability to identify a formulation exhibiting suitable performance for topical applications.

Particular emphasis was placed on the determination of the sol–gel transition temperature, which represents a critical quality attribute for thermoresponsive systems and is primarily governed by polymer concentration. A series of intermediate experimental trials was conducted to precisely define the gelation temperature and to achieve a formulation exhibiting phase transition behavior compatible with physiological conditions. Based on these preliminary investigations, the polymeric composition was optimized to ensure appropriate gel formation at body temperature while maintaining fluidity under refrigerated storage.

The final formulation, qualitatively composed of Poloxamer 407, PEG 1500, and the aqueous olive leaf extract, demonstrated desirable thermoresponsive characteristics, forming a stable gel at physiological temperature while maintaining a low-viscosity sol state under refrigerated conditions.

Although the exact quantitative composition of the optimized formulation is not disclosed due to ongoing intellectual property protection and commercialization considerations, all functional excipients and preparation principles are fully described in Section 5. The formulation parameters were systematically investigated within predefined compositional ranges to ensure reproducibility and robustness of the system. Based on these optimization studies, the final composition was fixed at 22% (*w*/*w*) Poloxamer 407 and 2% (*w*/*w*) PEG 1500, representing the optimal balance between thermoresponsive behavior and physicochemical stability. The use of defined concentration ranges during the development phase, followed by selection of a fixed optimized composition, provides a reproducible formulation framework. This approach ensures content uniformity, consistent gelation performance, and reliable experimental outcomes, thereby supporting the scientific validity and reproducibility of the formulation strategy.

The selected formulation exhibited reproducible physicochemical properties and consistent performance throughout the experimental evaluations.

The incorporation of oleuropein into the thermoresponsive polymeric matrix resulted in a formulation with appropriate mechanical strength, spreadability, and stability. These characteristics support its suitability for localized topical delivery while preserving the biological activity of the extract. The thermoresponsive formulation investigated in this study was obtained following a series of preliminary formulation optimization experiments aimed at achieving a stable liquid state at low temperature and a rapid sol–gel transition close to physiological conditions. In poloxamer-based systems, the sol–gel transition temperature is primarily governed by the polymer concentration and the interactions within the aqueous polymer network. Through iterative formulation screening, a polymer composition was selected that consistently produced a sol–gel transition at approximately 33 °C, as confirmed by rheological and vibroviscometric measurements. Due to ongoing intellectual property protection related to the formulation, the exact quantitative composition of the polymer matrix is not disclosed at this stage; however, the preparation procedure, thermoresponsive behavior, and physicochemical characteristics of the system are fully reported to allow interpretation of the formulation strategy and its functional performance.

### 2.2. Oleuropein Determination in the Sol–Gel Formulation

The homogeneous distribution of oleuropein within the sol–gel matrix was confirmed by HPLC analysis, indicating uniform incorporation of the olive leaf extract into the thermoresponsive polymeric system. The HPLC method employed for oleuropein quantification demonstrated excellent linearity under the applied chromatographic conditions, with a correlation coefficient exceeding 0.999. The regression equation was determined as *y* = 0.0824*x* + 0.0694, and the detector response was linear over the concentration range of 2.5–180 μg/mL.

The limits of detection (LOD) and quantification (LOQ) were determined to be 0.14 μg/mL and 0.43 μg/mL, respectively. These values correspond to the lowest concentration level of the standard solution that yielded a relative standard deviation (%RSD) of ≤2%. The LOD and LOQ were calculated according to the equations LOD = 3.3 × SD and LOQ = 10 × SD, where SD represents the standard deviation of the peak areas obtained from six replicate injections (n = 6) of the lowest concentration standard solution.

**Figure 1:**
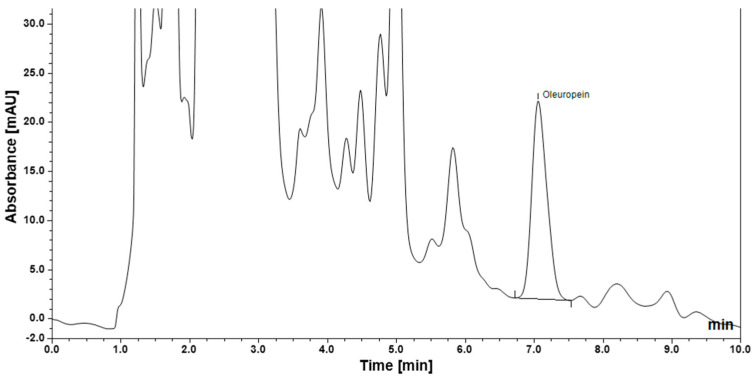
Oleuropein peak in sol–gel formulation.

Representative chromatograms of the oleuropein-containing sol–gel formulation and the placebo (blank) formulation are presented in Figure 1. The placebo formulation, prepared under identical conditions using purified water instead of olive leaf extract, exhibited no detectable peaks at the retention time corresponding to oleuropein. Quantitative analysis revealed an oleuropein concentration of 61.57 μg/mL in the sol–gel formulation.

**Figure 2:**
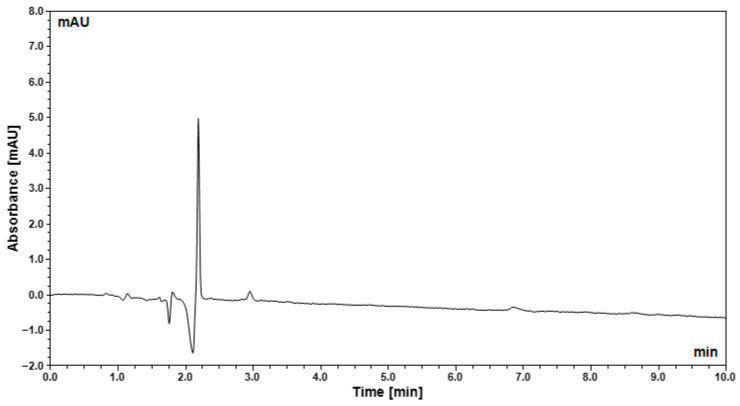
Representative blank chromatogram obtained under the optimized HPLC conditions.

The specificity of the method was evaluated by analyzing a blank solution under the same chromatographic conditions. The blank chromatogram revealed no detectable peaks at the retention time corresponding to oleuropein (RT = 7.23 min), indicating the absence of interference from solvents, reagents, or system-related contaminants (Figure 2).

### 2.3. Sol–Gel Transition Temperature

**Figure 3:**
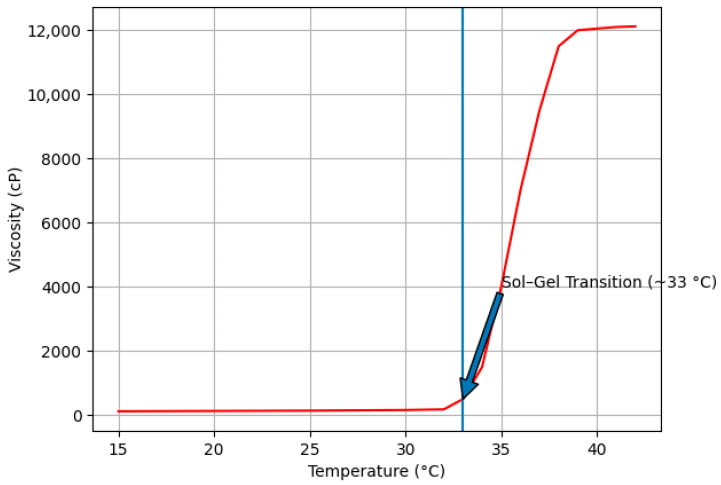
Viscosity temperature profile in heating profile.

The thermoresponsive sol–gel transition behavior of the formulation was evaluated using a vibro-viscometer (SV-10, A&D Instruments, Tokyo, Japan). The viscosity–temperature relationship during the controlled heating cycle is presented in Figure 3.

Upon gradual heating from room temperature to 42 °C, the formulation exhibited a characteristic thermally induced phase transition. A pronounced and rapid increase in viscosity was observed at approximately 33 °C, corresponding to the sol–gel transition temperature. This sharp viscosity elevation indicates the formation of a structured gel network associated with temperature-triggered micellar packing of Poloxamer 407.

**Figure 4:**
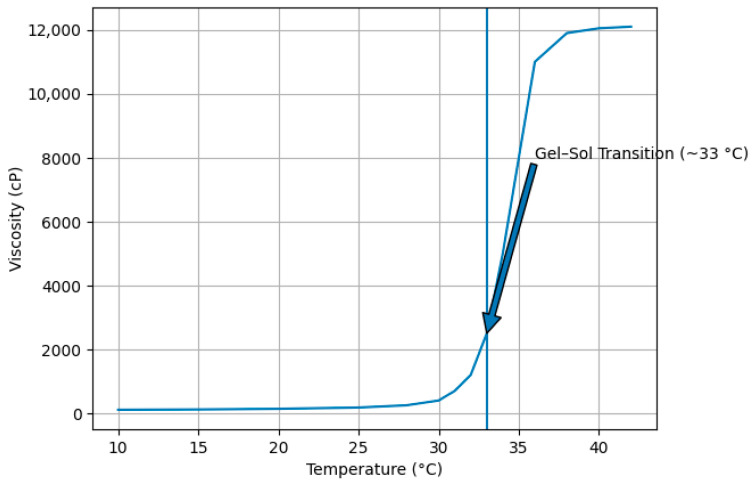
Viscosity temperature profile in cooling profile.

To assess the reversibility of the transition, the formulation was subsequently subjected to a controlled cooling cycle. As illustrated in Figure 4, a progressive decrease in viscosity was observed with decreasing temperature, and the system returned to its initial low-viscosity sol state.

The comparison of heating and cooling profiles confirms the thermoreversible nature of the sol–gel system. The absence of irreversible viscosity shifts or structural breakdown suggests that the gelation process is governed by reversible physical interactions rather than permanent chemical changes.

This thermoresponsive behavior is particularly advantageous for localized applications, as the formulation remains fluid during administration and undergoes rapid in situ gelation at physiological temperature, thereby enhancing residence time and surface coverage at the site of application.

### 2.4. Physicochemical Quality Control and Short-Term Stability Assessment

The physicochemical quality control parameters of the developed sol–gel formulation, including pH, density, electrical conductivity, refractive index, and viscosity, were evaluated over a three-month observation period (Table 1). The formulation exhibited a pH value of 6.9 ± 0.02, which falls within the physiologically acceptable range for topical application, supporting skin compatibility and formulation stability. The density was measured as 1.028 ± 0.001 g/mL, indicating uniform structural composition.

Electrical conductivity was determined to be 1935 ± 0.6 μS/cm, reflecting the ionic character of the system and suggesting consistent electrochemical behavior. The refractive index values ranged between 1.361 and 1.366, confirming optical clarity and homogeneity of the formulation. Viscosity measurements remained within a narrow range throughout the study, demonstrating the absence of significant structural instability.

No statistically significant variations (*p* > 0.05) were observed in any of the evaluated parameters during the three-month period, indicating that the formulation maintained its physicochemical integrity and performance characteristics under the tested storage conditions.

### 2.5. Mechanical Properties

The texture properties of the formulations, including hardness, adhesiveness, cohesiveness, resilience, and springiness, were evaluated. The results are summarized in Table 2.

Hardness (N) is defined as the maximum force required to achieve deformation during the first compression cycle and reflects the mechanical strength of the formulation. In topical gels, hardness is closely related to spreadability and may influence the residence time at the site of application.

Adhesiveness represents the work required to overcome the attractive forces between the probe and the sample surface, thereby indicating the adhesive performance of the system. Higher adhesiveness values suggest stronger interaction with biological surfaces, which may contribute to prolonged localization at the application site. Quantitatively, adhesiveness is calculated from the negative area of the force–distance curve obtained during probe withdrawal.

Cohesiveness describes the extent to which the formulation maintains its structural integrity under repeated mechanical deformation. It is expressed as the ratio of the work performed during the second compression cycle (A2) to that of the first compression cycle (A1).

Springiness refers to the ability of the gel to recover its original shape after deformation and reflects its elastic behavior. Resilience indicates the capacity of the sample to recover energy following the first compression cycle. These parameters collectively characterize the viscoelastic performance of the formulations [25,26,27].

### 2.6. Spreadability Analysis

Spreadability is a critical quality attribute of topically applied pharmaceutical formulations, as it directly affects ease of application, uniform distribution over the skin surface, and dose consistency, thereby influencing patient compliance [28]. Formulations with adequate spreadability facilitate smooth application with minimal shear stress, enabling homogeneous coverage of the target area. Improved spreading behavior may also enhance therapeutic performance by promoting consistent contact with the application site [29]. The spreadability results obtained for the tested formulations are presented in Table 3.

### 2.7. In Vitro Cell Culture Studies

In this study, the short-term biological effects of the oleuropein-enriched sol–gel were evaluated on L929 fibroblasts, and the formulation was assessed as non-cytotoxic over a wide concentration range. Oleuropein is a natural phenolic compound of olive leaf origin with reported antimicrobial and antioxidant activity and stands out among natural agents suitable for wound environments [23].

**Figure 5:**
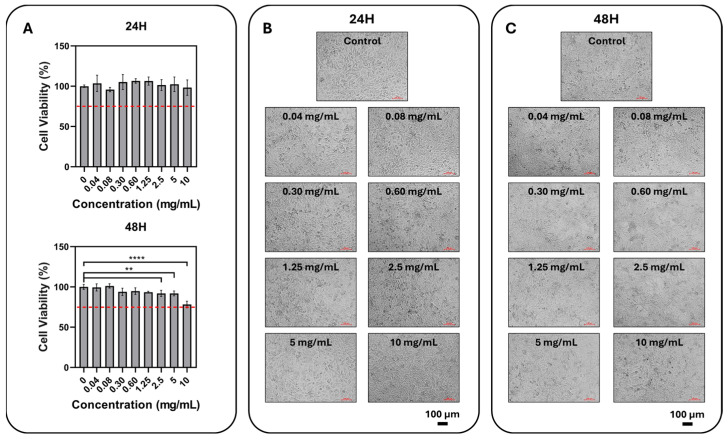
Effect of the lyophilized total formulation concentration on cell viability and morphology in L929 fibroblasts. (**A**) L929 cells were incubated with the formulation at the indicated final concentrations (0, 0.04, 0.08, 0.30, 0.60, 1.25, 2.5, 5, and 10 mg dry formulation/mL medium) for 24 and 48 h, and cell viability was calculated using the MTT test and compared to the control group. Data are presented as mean ± SD (n = 6). The red dashed line indicates the commonly used 70% viability threshold. Statistical analysis was performed using one-way ANOVA and Tukey’s post hoc multiple comparison test; significance levels are indicated by stars (ns: *p* ≥ 0.05, **: *p* < 0.01, ****: *p* < 0.0001). (**B**,**C**) Representative bright-field microscope images are given for each concentration after 24 h (**B**) and 48 h (**C**) (scale bar: 100 μm).

The 24- and 48 h MTT results (Figure 5) obtained with the lyophilized total formulation (0.04–10 mg/mL, expressed as mg dry formulation/mL medium) showed that cell viability remained close to the control across most tested concentrations. At 24 h, viability ranged from 95.80 ± 2.74% to 106.53 ± 2.80%, with the lowest value observed at 0.08 mg/mL and the highest at 0.60 mg/mL. At 48 h, viability ranged from 78.17 ± 4.11% to 101.24 ± 2.68%, with the lowest value observed in the 10 mg/mL group and the highest at 0.08 mg/mL. These findings indicate that the formulation was well tolerated over a broad concentration range, while prolonged exposure to the highest concentration resulted in a moderate reduction in metabolic activity. The similarity of cell morphologies to the control group suggests that this decrease in metabolic activity may be related to a mild stress response developing at high doses or a slowing of the proliferation/metabolism rate, rather than widespread/acute toxicity. In this respect, the decrease observed only at the highest dose and with longer exposure supports the idea that the formulation has a generally biocompatible safety window but that a threshold mechanism that may disrupt cell homeostasis at high doses is possible.

**Figure 6:**
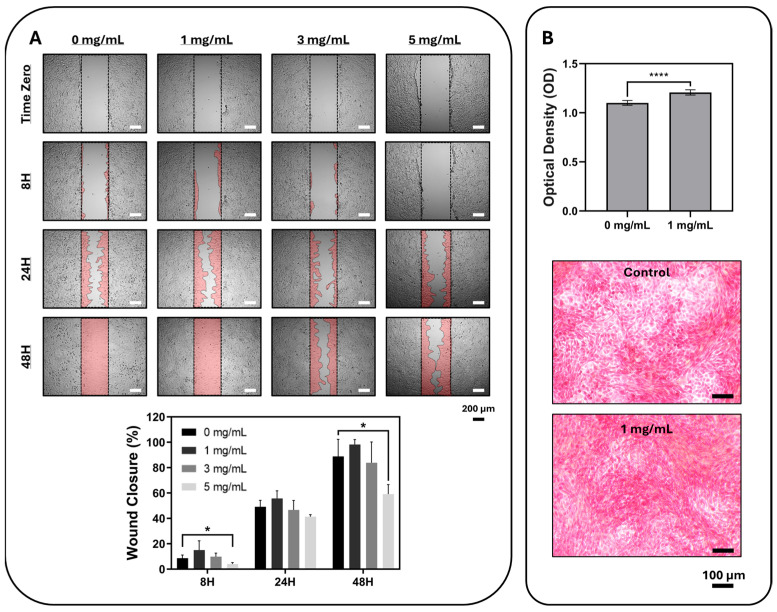
Evaluation of cell migration and cell-bound collagen deposition (Sirius Red S) in L929 fibroblasts. (**A**) Representative bright-field images of the artificial wound area created in the control (0 mg/mL) and selected concentration groups (1, 3, and 5 mg dry formulation/mL medium) at different time points are given. Marking of the wound area for image analysis is shown in red color, and quantitative wound closure (%) was calculated from ImageJ-measured wound area (data are presented as mean ± SD, n = 5 independent wound areas per group; statistical analysis was performed using one-way ANOVA, *: *p* < 0.05). (**B**) Matrix-associated staining was evaluated by Sirius Red and quantified by measuring the OD_540_ of the eluted dye. Values represent relative staining intensity and were not normalized to cell number or viability. Representative microscopic images of the control and 1 mg/mL groups stained with Sirius Red S are shown. All quantitative data are presented as mean ± SD, n = 16. Statistical analysis was performed using Student’s *t*-test. Significance levels are indicated with asterisks (****: *p* < 0.0001). Scale bars are provided on the images.

Scratch assay quantification based on ImageJ (version 1.54g)-measured wound area demonstrated a time-dependent increase in wound closure in all groups (Figure 6A). At 8 h, wound closure was 8.62 ± 2.49%, 15.02 ± 7.37%, 9.86 ± 2.79%, and 4.01 ± 1.22% for the 0, 1, 3, and 5 mg/mL groups, respectively, with the 5 mg/mL group showing significantly lower closure than the control (*p* = 0.0249). At 24 h, wound closure reached 49.11 ± 5.11%, 55.78 ± 6.01%, 46.68 ± 7.46%, and 41.30 ± 1.63%, respectively, and no statistically significant difference was observed relative to the control. At 48 h, closure further increased to 88.80 ± 13.53% in the control, 98.29 ± 3.83% at 1 mg/mL, 83.82 ± 16.46% at 3 mg/mL, and 59.15 ± 7.48% at 5 mg/mL; at this time point, the 5 mg/mL group again showed significantly lower wound closure than the control (*p* = 0.0116). Overall, the 1 mg/mL group displayed wound-closure behavior comparable to, and numerically higher than, the control, whereas 5 mg/mL delayed scratch closure despite remaining within a non-cytotoxic range in the MTT assay.

Although the FBS ratio is reduced, the closure of the scratch test is affected not only by migration but also by proliferation. Therefore, these findings were interpreted as a wound-closure response rather than as an isolated migration phenotype. A plausible explanation for this biphasic behavior is concentration-dependent redox modulation by the oleuropein-rich formulation, while lower concentrations may preserve a cellular environment favorable for repair, higher phenolic loading may begin to disturb reactive oxygen species (ROS)-sensitive signaling and cytoskeletal dynamics required for efficient closure. In line with this interpretation, the highest concentration also produced the greatest reduction in metabolic activity at 48 h in the MTT assay, supporting a sub-cytotoxic but functionally relevant effect on fibroblast behavior [30,31].

Based on the quantitative scratch assay findings, 1 mg/mL was selected for Sirius Red staining, as this concentration showed wound-closure behavior comparable to the control without evidence of impairment (Figure 6B). At the selected concentration, the formulation yielded a higher OD_540_ value than the control group in the Sirius Red assay, indicating a relative increase in collagen-associated staining intensity under the tested conditions. However, because the OD values were not normalized to cell number or viability, this finding was interpreted as a semi-quantitative difference in collagen-associated staining intensity rather than a definitive increase in collagen production on a per-cell basis. Therefore, the results do not allow a definitive conclusion regarding stimulation of colla-gen production. Further studies including normalization strategies and analysis of fibrosis- and matrix-remodeling-related markers, such as COL1A1, COL3A1, α-SMA, and TGF-β-associated pathways, would be needed to clarify whether this staining difference reflects beneficial matrix formation or other changes in the cellular microenvironment.

### 2.8. Evaluation of Biofilm Formation Inhibition

The antibiofilm activity of the olive leaf extract–incorporated sol–gel formulation was assessed over a concentration range of 50–400 μg/mL. At the lowest tested concentration (50 μg/mL), a slight numerical decrease in OD_570_ values was observed across all tested strains; however, this reduction was not statistically significant compared with the untreated control group (*p* > 0.05).

A statistically significant inhibition of biofilm formation was first detected at 100 μg/mL (*p* < 0.05), indicating the onset of a concentration-dependent antibiofilm effect. The inhibitory activity increased progressively with rising concentrations. At the highest concentration (400 μg/mL), the formulation exhibited maximal efficacy, reducing biofilm biomass by 73.7% for *S. aureus*, 63.8% for *E. coli*, and 66.7% for *P. aeruginosa* (Table 4).

### 2.9. Comparative Analysis of Planktonic Viability and Biofilm Recalcitrance

Assessment of planktonic growth revealed that the strongest effect among the three tested bacteria was observed against *S. aureus*; therefore, planktonic inhibition studies were primarily conducted on this species. The results showed that at different concentrations (100–400 μg/mL), the optical density at 600 nm (OD600) decreased, with a maximum inhibition of 15.2% observed at 400 μg/mL. To determine whether the reduction in biofilm biomass resulted from bactericidal effects or from specific interference with biofilm development, planktonic bacterial growth was evaluated in parallel. Spectrophotometric measurements at OD600 indicated that the olive leaf extract (OLE) exerted only a limited effect on planktonic cell proliferation (Table 5).

At the highest tested concentration (400 μg/mL), biofilm formation was markedly inhibited (60–74%), whereas planktonic cell density exhibited only a modest reduction (~15%) relative to the untreated control. These results suggest that the antibiofilm activity of OLE may involve a non-lethal mechanism, likely disrupting key stages of the biofilm development, such as initial surface attachment, the transition from reversible to irreversible adhesion, or modulation of extracellular polymeric substance (EPS) matrix formation, rather than causing generalized growth inhibition or bactericidal effects.

Such selective antibiofilm activity may be advantageous, as it targets biofilm-associated persistence while potentially minimizing the selective pressures commonly associated with the emergence of antimicrobial resistance.

### 2.10. Genotoxicity Assay

**Figure 7:**
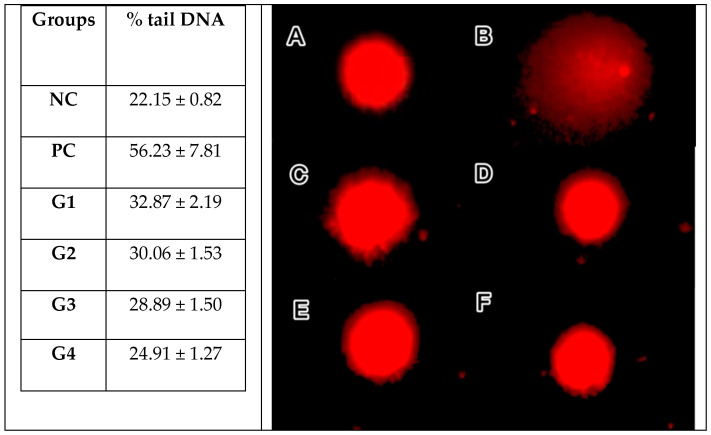
% Tail DNA values (mean ± SD) and representative comet micrographs (×40, EtBr staining) of study groups. (**A**) NC: negative control; (**B**) PC: positive control; (**C**) G1 (6.157 μg/mL): sol–gel formulation containing olive leaf extract; (**D**) G2 (3.0785 μg/mL): sol–gel formulation containing olive leaf extract; (**E**) G3 (1.539 μg/mL): sol–gel formulation containing olive leaf extract; (**F**) G4: sol–gel matrix without olive leaf extract (vehicle control).

The alkaline comet assay, also known as single-cell gel electrophoresis, was used to detect the genotoxic potential of the sol–gel formulation. %Tail DNA and representative comet images from each group are shown in Figure 7. The negative control (NC) exhibited the lowest mean % tail DNA, whereas the positive control (PC) showed the highest values, and a clear concentration-dependent increase between G1 to G3 and vehicle control (G4) confirms the appropriate assay performance.

The vehicle control showed a modest but statistically significant increase relative to the NC (*p* < 0.001); however, the difference in % tail DNA values compared to the PC group indicates no biologically meaningful increase in DNA strand breaks.

**Figure 8:**
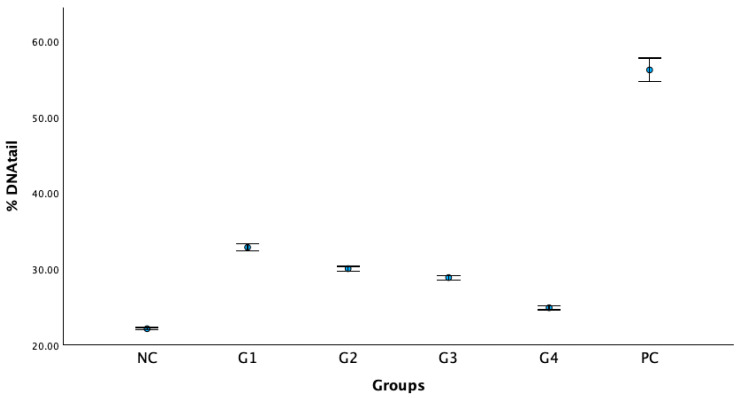
% tail DNA values of study groups (mean ± SD). NC: negative control; PC: positive control; G1 (6.157 μg/mL), G2 (3.0785 μg/mL), and G3 (1.539 μg/mL): sol–gel formulation containing olive leaf extract at different concentrations; G4: sol–gel matrix without olive leaf extract (vehicle control).

Although the observed increase in DNA damage was statistically significant, the magnitude of the effect was relatively small and occurred at concentrations far below cytotoxic levels. This pattern suggests the presence of low-level DNA damage rather than noticeable genotoxicity. Representative comet images for each group are presented in Figure 8.

## 3. Discussion

The present study describes the development and evaluation of a thermoresponsive sol–gel formulation incorporating an oleuropein-rich aqueous olive leaf extract. The formulation strategy was designed to address a fundamental limitation associated with natural bioactive compounds: insufficient residence time at the site of local administration. By integrating oleuropein into a temperature-triggered sol–gel formulation system, both physicochemical stability and biological functionality were targeted.

The thermoresponsive behavior observed in the present formulation is consistent with previously reported Poloxamer 407-based systems, where sol–gel transition typically occurs within the 30–35 °C range depending on polymer concentration and formulation composition [32,33]. The transition temperature detected in this study (~33 °C) falls within this optimal range, supporting its suitability for topical and mucosal applications. The sharp increase in viscosity near the transition temperature reflects temperature-induced micellar aggregation and packing, which is characteristic of poloxamer-based thermogelling systems. Furthermore, the reversibility of the viscosity–temperature profile indicates that gelation is governed by non-covalent micellar interactions rather than irreversible structural changes [20].

This thermoresponsive behavior is particularly advantageous for localized applications, as the formulation remains fluid during administration and rapidly forms a gel at physiological temperature, thereby enhancing residence time and surface coverage at the application site.

In the present system, PEG 1500 was incorporated as a supportive hydrophilic excipient rather than a primary thermoresponsive polymer. Polyethylene glycols are widely used to improve formulation consistency, wetting properties, and spreading behavior. In addition, PEG may influence the microstructure of poloxamer-based gels by modulating micellar organization and stabilizing the gel network, depending on its concentration and molecular weight. In this context, PEG 1500 was included to enhance formulation stability and application performance without altering the fundamental thermoresponsive mechanism governed by Poloxamer 407.

Regarding the phytochemical composition, the hydrophilic phenolic constituents of the olive leaf extract, including oleuropein, may interact with the aqueous microenvironment of the polymeric matrix. However, the viscosity–temperature profile remained consistent with typical poloxamer-based systems, suggesting that the primary determinant of thermoresponsive behavior is the polymer matrix itself. The phytochemical components are therefore considered to contribute mainly to biological activity rather than to the structural behavior of the system.

Physicochemical quality control parameters remained stable throughout the three-month observation period. The absence of statistically significant variations in pH, density, conductivity, refractive index, and viscosity suggests that the formulation maintained structural integrity and homogeneity under the tested storage conditions. The measured pH (~6.9) falls within the acceptable range for dermal compatibility, minimizing the risk of irritation and supporting biological tolerability.

Mechanical characterization demonstrated that the formulation possessed low hardness and favorable viscoelastic properties. Hardness, adhesiveness, cohesiveness, resilience, and springiness values collectively indicate a gel structure capable of maintaining integrity while allowing adequate spreading and tissue interaction. These parameters are critical for topical formulations, where excessive stiffness may hinder application, while insufficient cohesiveness may compromise retention.

Spreadability analysis further supported the applicability of the system. Appropriate firmness and work of shear values indicate that the formulation can be distributed uniformly with minimal mechanical resistance. This property is particularly important for wound environments, where homogeneous coverage directly influences therapeutic effectiveness.

Although additional characterization techniques such as mucoadhesion testing, in vitro release profiling, and accelerated stability studies could provide further insight into formulation performance, the primary objective of this work was to evaluate the thermoresponsive behavior and local biological activity of the oleuropein-enriched sol–gel system. The adhesiveness values obtained from texture profile analysis indicated an adequate interaction potential with biological surfaces, supporting localized retention of the formulation. Furthermore, the observed antibiofilm activity and favorable cellular responses suggest that the oleuropein-rich system remains functionally active under the tested conditions. Nevertheless, extended mucoadhesion characterization, release profiling, and accelerated stability studies may be considered in future investigations to support advanced formulation development and translational applications.

In the present study, in vitro release kinetics, diffusion, and permeation behavior were not investigated, which represents a limitation of the current work. However, the formulation was intentionally designed as a thermoresponsive topical system aimed at localized retention and surface-associated biological activity rather than systemic or transdermal delivery. In such systems, the therapeutic effect is primarily governed by residence time, matrix–tissue interaction, and sustained local exposure of bioactive compounds, rather than diffusion-driven transport across biological barriers.

Poloxamer 407-based thermoresponsive systems are known to form micellar structures and gel networks at physiological temperatures, which can modulate the mobility and release of incorporated compounds. Previous studies have demonstrated that poloxamer matrices may provide a diffusion-controlled or matrix-retained release profile depending on polymer concentration and formulation composition, often favoring prolonged local retention over rapid release [34,35]. In addition, the presence of hydrophilic excipients such as PEG may influence gel microstructure and water channel formation, potentially affecting molecular mobility within the matrix.

Within this context, the current study should be interpreted as a formulation and biological proof-of-concept demonstrating that the oleuropein-rich extract remains biologically active within the thermoresponsive matrix. Further investigations involving in vitro release kinetics, diffusion, and permeation models are currently ongoing and will be addressed in future studies to support advanced pharmaceutical development and comprehensive characterization of the system.

The three-month stability evaluation performed in this study was intended to provide preliminary information on the physicochemical integrity of the developed thermoresponsive sol–gel system during the experimental observation period. The absence of significant variations in pH, viscosity, density, electrical conductivity, and refractive index suggests that the formulation maintained structural homogeneity and stability under the tested storage conditions. Since the present work primarily focuses on formulation development and biological performance of a plant-derived topical system rather than full shelf life determination, the stability assessment was limited to short-term monitoring. Nevertheless, extended long-term or accelerated stability studies may be conducted in future investigations to further define the storage conditions and shelf life of the formulation.

Biological evaluation revealed a concentration-dependent response. The MTT assay demonstrated that the formulation was non-cytotoxic across a broad concentration range, with only a moderate reduction in metabolic activity at the highest tested dose following prolonged exposure. The preservation of fibroblast morphology at lower concentrations suggests acceptable cytocompatibility within the optimized safety window.

Scratch assay findings indicated that the formulation did not impair fibroblast migration at 1 mg/mL, whereas higher concentrations resulted in reduced migration rates. This dose-sensitive behavior may reflect the dual nature of phenolic compounds, which can exert protective antioxidant effects at moderate levels but may influence cellular signaling or redox balance at elevated concentrations. Such biphasic responses have been reported for several polyphenolic systems.

Sirius Red staining demonstrated enhanced collagen-associated responses at the selected non-inhibitory concentration. This observation may be associated with oleuropein’s reported modulation of extracellular matrix dynamics and tissue repair pathways. However, distinguishing between regenerative stimulation and potential fibrotic signaling requires further mechanistic investigation involving collagen subtype analysis and fibrosis-related markers.

The antibiofilm results represent a pivotal finding of this study. The oleuropein-enriched formulation significantly inhibited biofilm formation from 100 μg/mL, with maximal suppression observed at 400 μg/mL. Notably, the crystal violet assay primarily quantifies total biofilm biomass and does not provide direct information on biofilm architecture or bacterial viability within the matrix. This experimental setup is therefore intended to evaluate inhibition of biofilm formation during the early stages of bacterial adhesion and development, rather than eradication of mature, pre-formed biofilms. Consequently, the reductions in OD_570_ values should be interpreted as inhibition of biofilm formation rather than complete biofilm eradication. Importantly, planktonic bacterial viability was largely preserved, indicating that biofilm reduction was not primarily due to bactericidal effects.

This selective antibiofilm behavior suggests that oleuropein may interfere with early adhesion processes, quorum sensing pathways, or EPS matrix organization rather than inducing direct bacterial killing. Such a mechanism is particularly valuable in biofilm-dominated infections, where conventional antimicrobial approaches often fail due to diffusion barriers and tolerance phenotypes. By targeting biofilm formation rather than viability, the formulation may reduce selective pressure associated with resistance development [36,37].

The present findings are consistent with those reported by [38], who demonstrated that olive leaf extract exhibits antioxidant and anti-inflammatory properties and significantly reduces DNA damage in endothelial cells, as assessed by the comet assay.

The formulation developed in this study was primarily designed to support localized topical activity rather than systemic drug delivery. Therefore, the experimental evaluation focused on parameters directly related to local performance, including thermoresponsive gelation behavior, rheological properties, and biological responses such as antibiofilm activity and fibroblast compatibility. The sol–gel transition observed near physiological temperature (~33 °C) together with the measured mechanical properties suggests that the formulation can remain at the application site and maintain effective surface interaction. Within this context, the biological assays performed provide functional evidence that the phenolic constituents of the olive leaf extract remain locally bioactive. Consequently, the present study emphasizes local retention and biological functionality of the system rather than diffusion-driven release behavior typically investigated in transdermal or systemic drug delivery formulations. Overall, the integration of oleuropein into a thermoresponsive sol–gel formulation appears to provide a multifunctional system combining controlled delivery, mechanical suitability, cytocompatibility, and antibiofilm efficacy.

## 4. Conclusions

In this study, a thermoresponsive sol–gel formulation incorporating an oleuropein-rich aqueous olive leaf extract was successfully developed and characterized. The formulation exhibited a physiologically relevant sol–gel transition temperature, reversible thermogelation behavior, stable physicochemical properties, and appropriate mechanical and spreadability characteristics.

In vitro biological evaluations demonstrated acceptable cytocompatibility within the tested concentration range and indicated that optimized concentrations preserved fibroblast migration and supported collagen-associated responses. The formulation also showed concentration-dependent antibiofilm activity against representative Gram-positive and Gram-negative bacterial strains.

Notably, the selective inhibition of biofilm formation in the absence of strong bactericidal effects suggests a non-lethal antibiofilm mechanism, which may be advantageous for minimizing resistance-related risks. These findings highlight the potential of thermoresponsive sol–gel systems as effective formulations for natural bioactive compounds requiring localized retention and controlled release.

Within the experimental conditions applied, the compounds did not demonstrate genotoxic potential in the comet assay. Although minor increases in % tail DNA were observed, these changes were small in magnitude and not indicative of biologically meaningful genotoxicity. Therefore, the compounds may be considered suitable antimicrobial candidates for further optimization studies.

Collectively, the results support the applicability of oleuropein-enriched thermoresponsive sol–gel formulations as promising candidates for localized wound care and antimicrobial management strategies. Future studies should focus on mechanistic elucidation, controlled release kinetics, and in vivo validation to further define therapeutic potential.

## 5. Materials and Methods

### 5.1. Materials

Poloxamer 407 (Synperonic^®^ PE/F127) was obtained from Croda (Yorkshire, UK). It is characterized as a non-ionic triblock copolymer composed of poly(ethylene oxide) and poly(propylene oxide) segments in a PEO–PPO–PEO configuration. The polymer has an average molecular weight of about 12.6 kDa and a PEO:PPO ratio close to 70:30. PEG 1500 (pharma grade) was supplied by Tekkim (Istanbul, Türkiye).

Olive leaves (*Olea europaea* L.) utilized in this study were harvested from cultivated olive trees that are not under protection in Gömeç, Balıkesir, Türkiye, located in the Aegean region known for intensive olive production. The geographical coordinates of the sampling site were 39°25′43.3452″ N and 26°48′28.1988″ E. Olive leaves used for extraction were collected in November, a period reported in the literature to coincide with relatively high levels of phenolic compounds, particularly oleuropein, in olive foliage. To minimize environmental variability and potential dilution effects associated with rainfall, harvesting was performed on a dry day without precipitation. Leaves were collected from the same olive tree used in our previous experimental studies in order to maintain consistency in plant source and phytochemical profile. Collection was carried out during the afternoon hours, when phenolic compounds in olive leaves are reported to remain relatively stable during the daily metabolic cycle. After harvesting, the leaves were immediately transported to the laboratory and processed according to the extraction procedure described below.

Olive leaf samples were collected in limited quantities during routine maintenance of cultivated olive trees, strictly for research purposes. The collection process did not involve any form of commercial exploitation or large-scale harvesting. *Olea europaea* L. is not classified under any endangered or protected categories according to CITES regulations. For quantitative analysis, an oleuropein reference standard supplied by Extrasynthese was employed for calibration.

The L929 fibroblast cell line (ATCC CCL-1) was used for the in vitro studies. In cell culture studies, Dulbecco’s Modified Eagle Medium (DMEM, high glucose), Fetal Bovine Serum (FBS), Penicillin–Streptomycin, and Trypsin–EDTA were obtained from Thermo Fisher Scientific, Waltham, MA, USA. Ca^2+^/Mg^2+^-free phosphate-buffered saline (PBS) was from Capricorn ScientificGmbH, Ebsdorfergrund, Germany. Cell viability was assessed using MTT from BioMatik, Kitchener, ON, Canada, and DMSO from Merck, Darmstadt, Germany. For Sirius Red staining and solution preparation, ethanol, glacial acetic acid, picric acid, and NaOH were purchased from Isolab, Eschau, Germany, as well as formaldehyde and Sirius Red (Direct Red 80) from Sigma-Aldrich/Merck, Darmstadt, Germany, and HCl from Tekkim, Istanbul, Türkiye.

### 5.2. Preparation of the Formulation

Dried olive leaves were extracted in purified water (1:10 *w*/*v*) at room temperature under continuous agitation. Briefly, the dried leaves were dispersed in purified water and maintained under controlled conditions for a 10-day extraction period to obtain an oleuropein-rich hydrophilic extract. At the end of the extraction process, the mixture was filtered to remove insoluble plant residues, yielding a clear aqueous olive leaf extract [39].

For the preparation of the thermoresponsive sol–gel system, Poloxamer 407 (Synperonic^®^ PE/F127) and PEG 1500 were gradually incorporated into the olive leaf extract under continuous magnetic stirring. The polymers were added slowly to ensure homogeneous dispersion and to prevent agglomeration. The resulting mixture was maintained under gentle stirring until complete wetting of the polymeric components was achieved.

Subsequently, the formulation was stored at 4 °C for 24 h to allow full hydration and dissolution of Poloxamer 407, facilitating the formation of a uniform thermoresponsive sol phase. This cold conditioning step ensured complete polymer solubilization and stabilization of the sol–gel system prior to further characterization.

All formulations used in the experimental analyses were prepared following the same standardized preparation protocol. Independent batches were produced under identical experimental conditions, and each batch was subsequently subjected to physicochemical and biological characterization. The thermoresponsive behavior of the system was evaluated by monitoring the viscosity changes during controlled heating, allowing the determination of the sol–gel transition temperature. This approach ensured the reproducibility of the formulation performance across independently prepared samples.

### 5.3. Determination of Oleuropein in Sol–Gel Formulation

Oleuropein content in the developed sol–gel formulation was quantified using a high-performance liquid chromatography (HPLC) method previously developed and validated by our group [23]. To ensure methodological clarity and reproducibility, the principal chromatographic conditions are summarized below.

Chromatographic separation was performed using a mobile phase consisting of acetonitrile and 0.1% (*v*/*v*) formic acid in water (25:75, *v*/*v*). The flow rate was maintained at 1.0 mL/min, and the injection volume was set to 20 μL. The column temperature and autosampler temperature were controlled at 25 °C throughout the analysis. Each chromatographic run was completed within 10 min. Detection of oleuropein was carried out using a diode array detector (DAD) at 280 nm, corresponding to the absorption maximum of the phenolic compound. Before HPLC analysis, the sol–gel samples were diluted fourfold prior to injection into the chromatographic system using ultrapure water. The sol–gel samples were then vortexed for approximately half a minute to ensure complete homogeneous dissolution. Sample solutions were passed through a 0.45 μm nylon syringe filter, and each solution was analyzed in triplicate. The oleuropein content in the sol–gel sample was quantified by external standard calibration using the oleuropein standard solutions.

HPLC analyses were conducted using a Thermo Scientific UltiMate™ 3000 system (Thermo Scientific, Germering, Germany), equipped with a dual gradient pump (DGP-3600SD), an autosampler (WPS-300TSL), a thermostatted column compartment (TCC-3000SD), and a diode array detector (DAD-3000). Chromatographic separation was achieved on a reversed-phase Inertsil^®^ ODS-3 column (150 mm × 4.6 mm i.d., 5 μm particle size; GL Sciences, Tokyo, Japan).

Data acquisition, instrument control, and chromatographic processing were performed using Chromeleon™ 7.2 Chromatography Data System software (Thermo Fisher Scientific, Bremen, Germany).

To confirm method specificity and exclude potential interference from formulation excipients, a placebo (blank) formulation was prepared using purified water and auxiliary components without the olive leaf extract. The blank formulation was subjected to the same chromatographic analysis conditions. The absence of peaks at the retention time corresponding to oleuropein verified that the detected oleuropein signal originated exclusively from the olive leaf extract incorporated into the sol–gel formulation.

### 5.4. Determination of Sol–Gel Transition Temperature

The sol–gel transition temperature of the formulation was determined using a vibroviscometer (SV-10, A&D Instruments, Tokyo, Japan) operating on the tuning fork vibration principle. This technique enables real-time, highly sensitive viscosity measurements by monitoring the energy required to sustain constant-frequency oscillation of sensor plates immersed in the sample.

Viscosity measurements were conducted by gradually increasing the temperature of the formulation from room temperature to 42 °C under controlled conditions. The selected upper temperature limit (42 °C) was defined to encompass the maximum physiological temperature range relevant to potential clinical scenarios, including febrile conditions. The viscosity profile was recorded continuously throughout the heating cycle. For the rheological measurement, approximately 40 mL of the formulation was transferred to the measuring system and analyzed using the specified geometry under a controlled temperature ramp (°C/min) to evaluate the sol–gel transition behavior; the sample was not mechanically mixed during the measurement in order to avoid disturbance of the gelation process.

The sol–gel transition temperature was identified as the point at which a pronounced and rapid increase in viscosity occurred, corresponding to thermally induced gelation of the thermoresponsive system. To evaluate the reversibility of the phase transition, the formulation was subsequently cooled to room temperature while continuously monitoring viscosity changes. A progressive decrease in viscosity and restoration of the initial low-viscosity state confirmed the thermoreversible behavior of the sol–gel system.

### 5.5. Quality Control of the Sol–Gel Formulation

The developed sol–gel formulation was subjected to quality control evaluation through the determination of key physicochemical parameters. Density was measured using a calibrated pycnometer. The pH and electrical conductivity were determined using a multiparameter analytical instrument (Mettler Toledo, Hertfordshire, UK). The physicochemical characterization of the formulations was performed using a pH and conductivity meter (Hanna Instruments, Woonsocket, RI, USA), a refractometer (Bellingham + Stanley Ltd., Tunbridge Wells, UK) for refractive index determination.

In addition, the refractive index of the formulation was measured to assess optical uniformity and system consistency. The viscosity of the formulation at 25 °C was also determined to characterize the flow behavior under controlled temperature conditions.

All measurements were performed at room temperature unless otherwise specified and recorded in triplicate. Results are expressed as mean ± standard deviation to ensure analytical reliability and reproducibility.

### 5.6. Mechanical Properties

The mechanical properties of the optimized sol–gel formulation were quantitatively evaluated using a TA-XT Plus C Texture Analyser (Stable Micro Systems Ltd., Godalming, UK). The parameters assessed included hardness, adhesiveness, cohesiveness, resilience, and springiness.

Measurements were performed using a Perspex cylindrical probe (P/25P, 25 mm diameter). A two-cycle compression test (Texture Profile Analysis, TPA) was applied under the following conditions: pre-test speed of 2.0 mm/s, test speed of 2.0 mm/s, and post-test speed of 2.0 mm/s. A trigger force of 0.001 N was used. Samples were compressed to a depth of 10.0 mm, with a 10 s interval between consecutive compression cycles [25,40].

All measurements were conducted under controlled temperature conditions (37 ± 0.5 °C) and repeated in triplicate. Results are presented as mean ± standard deviation.

### 5.7. Spreadability

The spreadability of the sol–gel formulation was evaluated using a TA-XT Plus C Texture Analyser (Stable Micro Systems Ltd., Godalming, UK) equipped with a cone spreadability rig.

All measurements were performed in triplicate using gel samples equilibrated at 37 ± 0.5 °C. A sufficient amount of the formulation was carefully placed into the lower (female) cone assembly to avoid air entrapment. The upper (male) cone probe was driven downward to a defined distance of 23 mm at a controlled test speed of 3.0 mm/s, followed by a return movement at a post-test speed of 10.0 mm/s [25].

Spreadability parameters, including firmness, stickiness, work of shear, and work of adhesion, were calculated from the force–time profiles generated during the test.

### 5.8. In Vitro Cell Culture Studies

Evaluating developed pharmaceutical formulations under in vitro conditions is extremely important. In this study, the biocompatibility of the oleuropein-enriched sol–gel formulation was assessed using mouse fibroblasts, L929, as recommended in ISO 10993-5:2009 [41]. Subsequently, the effects of specific concentrations on cell migration and collagen production were examined via scratch assay and Sirius red staining, respectively. Cells were cultured in a 37 °C incubator containing 5% CO_2_ in standard high-glucose DMEM supplemented with 10% FBS and 1% penicillin-streptomycin. Once sufficient density was reached, cells were washed with Ca/Mg^2+^-free DPBS, removed with trypsin, and seeded for specific tests. For all in vitro experiments, test concentrations refer to the final concentration of the lyophilized total formulation reconstituted in culture medium and are expressed as mg dry formulation per ml medium (mg/mL).

#### 5.8.1. MTT Cytotoxicity Test

An MTT test was performed to determine the cytotoxicity of the developed formulation. The lyophilized total formulation was sterilized under UV light and reconstituted in complete medium at final concentrations of 0.04, 0.08, 0.30, 0.60, 1.25, 2.5, 5, and 10 mg dry formulation/mL medium. The control group received medium without formulation (0 mg/mL). Cells that reached sufficient confluence were removed with trypsin and plated into 96-well plates at a cell density of 8 × 10^3^ cells per well and allowed to adhere overnight in the incubator. Once a stable cell monolayer was established, the prepared formulations were introduced into the wells by replacing the culture medium with 100 μL of formulation-containing medium. Wells receiving only fresh culture medium were designated as controls. At predetermined time points (24 and 48 h), cellular morphology was documented using representative imaging. For viability assessment, MTT reagent was added directly to each well (10 μL; final concentration adjusted to 0.5 mg/mL in the medium), and the plates were incubated for 4 h to allow formazan formation. Following incubation, the supernatant was carefully aspirated, and the intracellularly formed formazan crystals were solubilized by the addition of 100 μL DMSO per well. Complete dissolution was ensured by gentle orbital shaking for approximately 5 min. The optical density was subsequently measured at 570 nm using a microplate spectrophotometer (BMG LABTECH SPECTROstar^®^ Nano, Ortenberg, Germany). Relative cell viability was quantified based on the corresponding equation [20]:

$$Cell\;Viability\;\left(\%\right)=\frac{{OD}_{570treatment}}{{OD}_{570control}}\times 100$$

Here, *OD*_570*treatment*_ is the absorbance of the formulation groups at 570 nm; *OD*_570*control*_ is the absorbance of the control group at 570 nm.

#### 5.8.2. Scratch Assay (Cell Migration)

Cell migration was investigated using an in vitro scratch assay to assess the wound-healing potential of the developed sol–gel system. Based on the cytocompatibility results obtained from the MTT assay, three non-toxic concentrations of the lyophilized formulation (1, 3, and 5 mg/mL, expressed as dry weight in culture medium) were selected for further evaluation. Test samples were freshly prepared in culture medium supplemented with 5% fetal bovine serum (FBS), instead of the standard 10% FBS. For the assay, 8 × 10^4^ cells were seeded into each well of 24-well plates and allowed to grow until a uniform and confluent monolayer was formed. A linear scratch was then introduced across the cell layer using a sterile 200 μL pipette tip to mimic a wound gap. Detached cells and residual debris were removed by gently rinsing the wells with phosphate-buffered saline (PBS). Subsequently, the treatment media containing the formulations were added, while control wells received medium supplemented with 5% FBS only. The plates were maintained under standard incubation conditions (37 °C, 5% CO_2_). Wound closure and cell migration were monitored by capturing images at defined time points (0, 8, 24, and 48 h) from consistent locations using an inverted microscope system (Zeiss Observer Z1 equipped with a CO_2_ incubation unit, Carl Zeiss, Jena, Germany) [20]. For each scratch region, images were captured from the same predefined coordinates at each time point so that the same wound area was followed throughout the experiment. Wound closure was quantified using ImageJ software (NIH, Bethesda, MD, USA) by measuring the wound area at each time point [42]. The results were expressed as percentage wound closure, calculated according to the following equation [43]:

$$Wound\;Closure\;\left(\%\right)=\;\frac{T_{0}-\;T_{x}}{T_{0}}\;\times 100$$

where T_0_ is the wound area at 0 h, and T_x_ is the wound area at the relevant time point. A total of five independent wound areas per group (n = 5) were analyzed. Since no proliferation inhibitor was used, the scratch assay results were interpreted as a wound-closure response reflecting the combined contribution of cell migration and proliferation [44].

#### 5.8.3. Sirius Red Staining

Cell-associated collagen deposition was evaluated using a plate-based colorimetric method with Sirius Red staining. L929 fibroblasts were seeded into a 96-well plate at a concentration of 5 × 10^4^ cells per well. After 24 h, the medium was changed to DMEM containing 1% FBS and incubated for another 24 h to ensure complete confluence. A concentration of 1 mg dry formulation/mL medium, showing similar results to the control in the migration test, was selected and administered to the cells in FBS-free medium. The control group received standard FBS-free medium. After 48 h of incubation, the medium was removed; the cells were washed with PBS and fixed in Kahle solution (26% ethanol, 3.7% formaldehyde, 2% glacial acetic acid) at room temperature for 15 min. After fixation, the wells were washed again with PBS and then stained with 0.1% Sirius Red (Direct Red 80) in 1% picric acid by incubation at room temperature for 1 h. After staining, the wells were washed with 0.1 M HCl, and collagen deposition before elution was visualized under a microscope in bright-field mode. To elute the bound dye, 100 μL of 0.1 M NaOH was added to each well, the eluate was transferred to a clean 96-well plate, and the absorbance was measured at 540 nm. NaOH was used as a blank [45].

### 5.9. Extraction of Phenolic Compounds from the Sol–Gel Formulation

To evaluate the phenolic profile associated with the developed sol–gel system, phenolic compounds were extracted from the formulation using a solvent extraction procedure.

Briefly, a defined amount of the sol–gel formulation was dispersed in a methanol/water mixture (80:20, *v*/*v*) to enable efficient recovery of hydrophilic phenolic constituents. The mixture was vortexed vigorously to ensure complete disruption of the gel matrix and adequate solvent interaction.

The resulting dispersion was centrifuged at 4000 rpm for 10 min at room temperature to facilitate phase separation. The supernatant containing the extracted phenolic compounds was carefully collected. The organic solvent was removed under reduced pressure using a rotary evaporator (Rotavapor R-300, Büchi, Flawil, Switzerland) at 40 °C.

The obtained phenolic residue was reconstituted in sterile deionized water, filtered through a 0.22 μm membrane filter, and stored at −20 °C until further analysis and antibiofilm testing.

### 5.10. Bacterial Strains and Inoculum Preparation

The antibiofilm activity of the extracted phenolic fraction was evaluated against three reference bacterial strains: *Staphylococcus aureus* (ATCC 25923), *Escherichia coli* (ATCC 25922), and *Pseudomonas aeruginosa* (ATCC 27853) [12,13]. These strains were selected based on their well-documented biofilm-forming capacity and frequent use in antimicrobial and antibiofilm studies.

Stock cultures were maintained on Tryptic Soy Agar (TSA) and routinely subcultured in Tryptic Soy Broth (TSB) at 37 °C. Strain identity was verified using matrix-assisted laser desorption/ionization time-of-flight mass spectrometry (MALDI-TOF MS) (Microflex LT, Bruker Daltonics, Bremen, Germany).

Prior to each experiment, overnight cultures were refreshed to obtain actively growing bacterial cells. The inoculum density was adjusted spectrophotometrically to an optical density at 600 nm (OD_600_), corresponding to approximately 10^8^ CFU/mL, to ensure uniform bacterial loading across experimental groups.

### 5.11. Microtiter Plate Biofilm Assay

The antibiofilm activity of the formulations was evaluated using the microtiter plate crystal violet assay with slight modifications [46]. The standardized bacterial suspension (10^8^ CFU/mL) was diluted 1:100 in fresh tryptic soy broth (TSB) prior to inoculation of the microtiter plates.

Aliquots of the diluted bacterial suspension were inoculated into sterile 96-well microtiter plates in the presence of the test formulations.

The plates were incubated at 37 °C for 24 h under static conditions to allow bacterial adhesion and biofilm formation. This experimental setup was designed to evaluate inhibition of biofilm formation during the early stages of bacterial adhesion and development rather than the eradication of mature pre-formed biofilms.

The extracted phenolic fraction was tested at final concentrations of 50, 100, 200, and 400 μg/mL to evaluate concentration-dependent effects on biofilm formation. Wells containing bacterial suspension without treatment served as negative controls, while wells containing only sterile growth medium were used as blanks.

All experiments were performed in triplicate, and the results were expressed as mean ± standard deviation.

### 5.12. Biofilm Biomass Quantification via Crystal Violet Staining

Following incubation, the wells were gently washed three times with sterile phosphate-buffered saline (PBS, pH 7.4) to remove planktonic cells while preserving the biofilm matrix. The adherent biomass was fixed and subsequently stained with 0.1% (*w*/*v*) crystal violet solution for 15 min at room temperature. Excess stain was removed by washing thoroughly with distilled water, and the matrix-bound crystal violet was solubilized using 95% ethanol.

The absorbance of the solubilized dye was measured at 570 nm using a spectrophotometric microplate reader (Multiskan GO, Thermo Fisher Scientific, Waltham, MA, USA) to quantify total biofilm biomass.

### 5.13. Genotoxicity Assay

#### 5.13.1. Study Group

A total of six experimental groups were included in the study. Isolated human peripheral blood lymphocytes were exposed to three concentrations of the sol–gel formulation (6.157, 3.0785, and 1.539 μg/mL), corresponding to 61.57 μg/mL olive leaf extract within the formulation, and these groups were designated as G1–G3. A sol–gel matrix lacking olive leaf extract served as the vehicle control (G4). Hydrogen peroxide (H_2_O_2_, 10 mM) was used as the positive control (PC), Phosphate-buffered saline (PBS) without any test material was used as the negative control (NC) to determine the baseline level of DNA damage. All treatment groups were incubated for 30 min at 37 °C, whereas the positive control group was exposed for 7 min under the same temperature conditions.

#### 5.13.2. Alkaline Comet Assay (Single Cell Gel Electrophoresis)

Approximately 0.5 mL peripheral blood was collected into heparinized tubes. Before the blood sample collection, ethical approval was obtained for this study. Histopaque 1077 density gradient was used for the isolation of lymphocytes (250 g, +4 °C, 10′). Trypan blue was used to evaluate cell viability by exclusion. In all analyzed samples, the proportion of dye-excluding (viable) cells consistently exceeded 90%.

The alkaline comet assay has been performed according to Singh et al. with slight modifications [47]. All cells were embedded in 0.5% low-melting agarose (LMA) on microscope slides that were previously coated with 0.5% high-melting agarose (HMA) after incubation with all studied groups. After gently removing coverslips, all slides were immersed into a cold lysing solution (2.5 M NaCl, 100 mM Na2EDTA, 10 mM Tris, pH 10). The post-lysis procedures were performed in the dark to avoid additional DNA damage. Following lysis, slides were left in electrophoresis solution (300 mM NaOH, 1 mM EDTA, pH 13) for 20 min to denature and unwind. Then electrophoresis was conducted at 15 V for 20 min at 300 mA. The slides were rinsed three times for 5 min each with neutralization buffer (0.4 M Tris buffer, pH 7.5) to eliminate residual alkali. Finally, the slides were fixed with 50%, 75%, and absolute ethanol and were kept at room temperature to dry [48].

#### 5.13.3. Slide Scoring and Statistical Analysis

Ethidium bromide (EtBr, 20 μL/mL) was used for each slide for image analysis at ×40 magnification under a fluorescent microscope (BAB, Ankara, Türkiye) equipped with a 546 nm excitation filter and 590 nm barrier filter. One hundred cells were counted and scored by BAB Fluorescent Microscope with the BAB Bs200ProP/BsComet DNA Comet Assay Image Analyzer program for each study group. Welch’s ANOVA was used to analyze group differences due to heterogeneity of variances, and Tamhane’s T2 post hoc test was applied for pairwise comparisons between groups. A *p*-value of ≤0.05 was considered statistically significant. The statistical software SPSS (ver. 23.0) was used to analyze the data.

### 5.14. Statistical Analysis

All assays were conducted in triplicate across at least three independent experimental runs. Data are presented as mean ± standard deviation (SD). Statistical significance was evaluated using one-way Analysis of Variance (ANOVA) followed by Tukey’s post hoc test. All statistical analyses and graphical representations were performed using GraphPad Prism software (version 9.0, GraphPad Software, San Diego, CA, USA). A *p*-value of <0.05 was established as the threshold for statistical significance.

## Figures and Tables

**Table 1:** Quality control parameters of the sol–gel formulation for 3 months.

Parameter	Initial	Month 1	Month 2	Month 3	Mean ± SD	%RSD
pH	6.90	6.89	6.91	6.90	6.90 ± 0.01	0.14
Density (g/mL)	1.028	1.027	1.029	1.028	1.028 ± 0.001	0.10
Conductivity (μS/cm)	1935	1934	1936	1935	1935 ± 0.6	0.04
Refractive Index	1.362	1.361	1.364	1.366	Stable range	—
Viscosity (cP at 25 °C)	218	221	216	219	218.5 ± 2.08	0.95
pH	6.90	6.89	6.91	6.90	6.90 ± 0.01	0.14

**Table 2:** Results of mechanical tests.

Hardness (g)	Adhesiveness (g·s)	Cohesiveness	Resilience (%)	Springiness (%)
0.621 ± 0.018	−9.328 ± 1.780	0.871 ± 0.019	19.473 ± 3.283	98.603 ± 0.952

**Table 3:** Results of the spreadability test.

Firmness (g)	Work of Shear (g·s)	Stickiness (g)	Work of Adhesion (g·s)
140.31 ± 8.28	87.98 ± 3.76	−203.49 ± 11.55	−28.48 ± 0.55

**Table 4:** Quantitative assessment of biofilm formation OD_570_ in the presence of varying olive leaf extract-incorporated sol–gel formulations.

Concentration (μg/mL)	*S. aureus*	*E. coli*	*P. aeruginosa*
Control (0)	1.18 ± 0.09 ^ns^	1.05 ± 0.07 ^ns^	1.32 ± 0.11 ^ns^
50	1.06 ± 0.08 ^ns^	0.96 ± 0.07 ^ns^	1.21 ± 0.10 ^ns^
100	0.71 ± 0.06 *	0.65 ± 0.06 *	0.78 ± 0.08 *
200	0.48 ± 0.05 *	0.52 ± 0.04 *	0.59 ± 0.06 *
400	0.31 ± 0.04 *	0.38 ± 0.03 *	0.44 ± 0.05 *

Values represent mean ± SD (n = 3). ns: non-significant; *: significant differences (*p* < 0.05) compared to the control group.

**Table 5:** Impact of olive oil extract on planktonic cell density OD600.

Concentration (μg/mL)	Planktonic Growth of *S. aureus* (OD600)	Inhibition (%)
Control (0)	0.92 ± 0.06	-
100	0.88 ± 0.05	4.3%
200	0.83 ± 0.04	9.8%
400	0.78 ± 0.05	15.2%

## Data Availability

The data presented in this study are available on reasonable request from the corresponding author.

## Abbreviations

The following abbreviations are used in this manuscript:

ANOVA	Analysis of Variance
ATCC	American Type Culture Collection
CFU	Colony Forming Unit
CITES	Convention on International Trade in Endangered Species
COL1A1	Collagen Type I Alpha 1 Chain
COL3A1	Collagen Type III Alpha 1 Chain
CO_2_	Carbon Dioxide
DAD	Diode Array Detector
DMEM	Dulbecco’s Modified Eagle Medium
DGP	Dual Gradient Pump
DMSO	Dimethyl Sulfoxide
DPBS	Dulbecco’s Phosphate-Buffered Saline
ECM	Extracellular Matrix
ELISA	Enzyme-Linked Immunosorbent Assay
EPS	Extracellular Polymeric Substance
FBS	Fetal Bovine Serum
HPLC	High Performance Liquid Chromatography
ISO	International Organization for Standardization
NaOH	Sodium Hydroxide
PBS	Phosphate-Buffered Saline
TPA	Texture Profile Analysis
TSA	Tryptic Soy Agar
TSB	Tryptic Soy Broth
WPS	Well Plate Sampler

## References

[1] Nediani C., Ruzzolini J., Romani A., Calorini L. (2019). Oleuropein, a Bioactive Compound from *Olea europaea* L., as a Potential Preventive and Therapeutic Agent in Non-Communicable Diseases. Antioxidants.

[2] Asmaey M.A., Elsoghiar A.A.M., Shaaban M., Moharram A.M., El-Gaby M.S.A. (2024). Phenolics and Other Structural Compounds from Leaves of *Olea europaea* L.: Extraction Techniques and Pharmacological Activities. Chem. Afr..

[3] Bilgin M., Şahin S. (2013). Effects of geographical origin and extraction methods on total phenolic yield of olive tree (*Olea europaea*) leaves. J. Taiwan Inst. Chem. Eng..

[4] Mechri B., Tekaya M., Hammami M., Chehab H. (2020). Effects of drought stress on phenolic accumulation in greenhouse-grown olive trees (*Olea europaea*). Biochem. Syst. Ecol..

[5] Zakraoui M., Hannachi H., Pasković I., Vidović N., Polić Pasković M., Palčić I., Major N., Goreta Ban S., Hamrouni L. (2023). Effect of Geographical Location on the Phenolic and Mineral Composition of Chetoui Olive Leaves. Foods.

[6] Ansari M., Kazemipour M., Fathi S. (2011). Development of a simple green extraction procedure and HPLC method for determination of oleuropein in olive leaf extract applied to a multi-source comparative study. J. Iran. Chem. Soc..

[7] Goldsmith C.D., Vuong Q.V., Stathopoulos C.E., Roach P.D., Scarlett C.J. (2014). Optimization of the Aqueous Extraction of Phenolic Compounds from Olive Leaves. Antioxidants.

[8] Monteleone J.I., Sperlinga E., Siracusa L., Spagna G., Parafati L., Todaro A., Palmeri R. (2021). Water as a Solvent of Election for Obtaining Oleuropein-Rich Extracts from Olive (*Olea europaea*) Leaves. Agronomy.

[9] El-Sayed N.R., Samir R., Jamil M.A.-H.L., Ramadan M.A. (2020). Olive Leaf Extract Modulates Quorum Sensing Genes and Biofilm Formation in Multi-Drug Resistant *Pseudomonas aeruginosa*. Antibiotics.

[10] Guo W., Xu Y., Yang Y., Xiang J., Chen J., Luo D., Xie Q. (2023). Antibiofilm Effects of Oleuropein against Staphylococcus aureus: An In Vitro Study. Foods.

[11] Esfandiary M.A., Khosravi A.R., Asadi S., Nikaein D., Hassan J., Sharifzadeh A. (2024). Antimicrobial and anti-biofilm properties of oleuropein against Escherichia coli and fluconazole-resistant isolates of Candida albicans and Candida glabrata. BMC Microbiol..

[12] Flemming H.-C., Wingender J., Szewzyk U., Steinberg P., Rice S.A., Kjelleberg S. (2016). Biofilms: An emergent form of bacterial life. Nat. Rev. Microbiol..

[13] Hall C.W., Mah T.F. (2017). Molecular mechanisms of biofilm-based antibiotic resistance and tolerance in pathogenic bacteria. FEMS Microbiol. Rev..

[14] Ciofu O., Moser C., Jensen P.Ø., Høiby N. (2022). Tolerance and resistance of microbial biofilms. Nat. Rev. Microbiol..

[15] Lu L., Hu W., Tian Z., Yuan D., Yi G., Zhou Y., Cheng Q., Zhu J., Li M. (2019). Developing natural products as potential anti-biofilm agents. Chin. Med..

[16] Alavi S.E., Raza A., Gholami M., Giles M., Al-Sammak R., Ibrahim A., Ebrahimi Shahmabadi H., Sharma L.A. (2022). Advanced Drug Delivery Platforms for the Treatment of Oral Pathogens. Pharmaceutics.

[17] Ryall C., Duarah S., Chen S., Yu H., Wen J. (2022). Advancements in Skin Delivery of Natural Bioactive Products for Wound Management: A Brief Review of Two Decades. Pharmaceutics.

[18] Pagano C., Giovagnoli S., Perioli L., Tiralti M.C., Ricci M. (2020). Development and characterization of mucoadhesive-thermoresponsive gels for the treatment of oral mucosa diseases. Eur. J. Pharm. Sci..

[19] Bejenaru L.E., Segneanu A.-E., Bejenaru C., Bradu I.A., Vlase T., Herea D.-D., Văruţ M.C., Bălăşoiu R.M., Biţă A., Radu A. (2025). Thermoresponsive gels with rosemary essential oil: A novel topical carrier for antimicrobial therapy and drug delivery applications. Gels.

[20] Alparslan L., Torkay G., Bal-Öztürk A., Köksal Karayıldırım Ç., Özdemir S. (2025). Smart Thermoresponsive Sol–Gel Formulation of Polyhexanide for Rapid and Painless Burn and Wound Management. Polymers.

[21] Asghariazar V., Vahidian F., Karimi A., Abbaspour-Ravasjani S., Mansoori B., Safarzadeh E., Laranjo M. (2024). The Role of Oleuropein, Derived from Olives, in Human Skin Fibroblast Cells: Investigating the Underlying Molecular Mechanisms of Cytotoxicity and Antioxidant and Anti-Inflammatory Activities. Int. J. Clin. Pract..

[22] Allaw M., Manca M.L., Gómez-Fernández J.C., Pedraz J.L., Terencio M.C., Sales O.D., Nacher A., Manconi M. (2021). Oleuropein Multicompartment Nanovesicles Enriched with Collagen as A Natural Strategy for The Treatment of Skin Wounds Connected with Oxidative Stress. Nanomedicine.

[23] Alparslan L., Özdemir S., Karacan B., Tutar Ö.F., Doğan T., Akar R.O., Yıldırım E.G., Erdoğan N. (2026). Formulation and Characterization of an Oleuropein-Enriched Oral Spray Gel: Microbiological Performance and In Ovo Histopathological Safety. Pharmaceutics.

[24] Collins A., Møller P., Gajski G., Vodenková S., Abdulwahed A., Anderson D., Bankoglu E.E., Bonassi S., Boutet-Robinet E., Brunborg G. (2023). Measuring DNA modifications with the comet assay: A compendium of protocols. Nat. Protoc..

[25] Çağlar E.Ş., Güven G.K., Okur N.Ü. (2023). Preparation and characterization of carbopol based hydrogels containing dexpanthenol. J. Fac. Pharm. Ank. Univ..

[26] Karakucuk A., Tort S., Han S., Oktay A.N., Celebi N. (2021). Etodolac nanosuspension based gel for enhanced dermal delivery: In vitro and in vivo evaluation. J. Microencapsul..

[27] Erel-Akbaba G., Akbaba H., Keselik E., Bahceci S.A., Senyigit Z., Temiz T.K. (2022). Octaarginine functionalized nanoencapsulated system: In vitro and in vivo evaluation of bFGF loaded formulation for wound healing. J. Drug Deliv. Sci. Technol..

[28] Al-Barghouthy E.Y., Hamed S., Mehyar G.F., AlKhatib H.S. (2025). Comparative Evaluation of Spreadability Measurement Methods for Topical Semisolid Formulations/A Scoping Review. Gels.

[29] Tomić I., Miočić S., Pepić I., Šimić D., Filipović-Grčić J. (2021). Efficacy and safety of azelaic acid nanocrystal-loaded in situ hydrogel in the treatment of acne vulgaris. Pharmaceutics.

[30] Balachandran A., Siyumbwa S.N., Froemming G.R., Beata M.-M., Małgorzata J., Lavilla C.A., Billacura M.P., Okechukwu P.N. (2023). In vitro antioxidant and fibroblast migration activities of fractions eluded from dichloromethane leaf extract of *Marantodes pumilum*. Life.

[31] Merecz-Sadowska A., Sitarek P., Kucharska E., Kowalczyk T., Zajdel K., Cegliński T., Zajdel R. (2021). Antioxidant Properties of Plant-Derived Phenolic Compounds and Their Effect on Skin Fibroblast Cells. Antioxidants.

[32] Bakhrushina E.O., Novozhilova E.V., Shumkova M.M., Pyzhov V.S., Nikonenko M.S., Bardakov A.I., Demina N.B., Krasnyuk I.I., Krasnyuk I.I. (2023). New Biopharmaceutical Characteristics of In Situ Systems Based on Poloxamer 407. Gels.

[33] Akash M.S., Rehman K. (2015). Recent progress in biomedical applications of Pluronic (PF127): Pharmaceutical perspectives. J. Control Release.

[34] Dumortier G., Grossiord J.L., Agnely F., Chaumeil J.C. (2006). A Review of Poloxamer 407 Pharmaceutical and Pharmacological Characteristics. Pharm. Res..

[35] Ruel-Gariépy E., Leroux J.-C. (2004). In situ-forming hydrogels—Review of temperature-sensitive systems. Eur. J. Pharm. Biopharm..

[36] Leouifoudi I., Harnafi H., Zyad A. (2015). Olive Mill Waste Extracts: Polyphenols Content, Antioxidant, and Antimicrobial Activities. Adv. Pharmacol. Sci..

[37] Sudjana A.N., D’Orazio C., Ryan V., Rasool N., Ng J., Islam N., Riley T.V., Hammer K.A. (2009). Antimicrobial activity of commercial *Olea europaea* (olive) leaf extract. Int. J. Antimicrob. Agents.

[38] Burja B., Kuret T., Janko T., Topalović D., Živković L., Mrak-Poljšak K., Spremo-Potparević B., Žigon P., Distler O., Čučnik S. (2019). Olive Leaf Extract Attenuates Inflammatory Activation and DNA Damage in Human Arterial Endothelial Cells. Front. Cardiovasc. Med..

[39] Otero D.M., Oliveira F.M., Lorini A., Antunes B.d.F., Oliveira R.M., Zambiazi R.C. (2020). Oleuropein: Methods for extraction, purifying and applying. Rev. Ceres.

[40] Çağlar E.Ş., Ayyıldız K.N., Doğanay D., Özkanca C., Saldamlı E., Abudayyak M., Okur N.Ü. (2025). Formulation, Characterization and In vitro-Ex Vivo Evaluation of Moxifloxacin Loaded Poloxamer and Methyl Cellulose Based In Situ Gel for Enhanced Ocular Delivery. J. Drug Deliv. Sci. Technol..

[41] (2009). Biological Evaluation of Medical Devices—Part 5: Tests for In Vitro Cytotoxicity.

[42] Suarez-Arnedo A., Torres Figueroa F., Clavijo C., Arbeláez P., Cruz J.C., Muñoz-Camargo C. (2020). An image J plugin for the high throughput image analysis of in vitro scratch wound healing assays. PLoS ONE.

[43] Kendir G., Güleç M., Öztürk A.B., Torkay G., Muhammed M.T., Olgun A., Köroğlu A. (2025). Biological and Phytochemical Insights Into *Opuntia ficus-indica* (L.) Mill: Cytotoxic, Wound-Healing, and Anti-Aging Potentials. Food Sci. Nutr..

[44] Jonkman J.E., Cathcart J.A., Xu F., Bartolini M.E., Amon J.E., Stevens K.M., Colarusso P. (2014). An introduction to the wound healing assay using live-cell microscopy. Cell Adh. Migr..

[45] Szász C., Pap D., Szebeni B., Bokrossy P., Őrfi L., Szabó A.J., Vannay Á., Veres-Székely A. (2023). Optimization of Sirius Red-Based Microplate Assay to Investigate Collagen Production In Vitro. Int. J. Mol. Sci..

[46] Stepanović S., Vuković D., Hola V., Di Bonaventura G., Djukić S., Cirković I., Ruzicka F. (2007). Quantification of biofilm in microtiter plates: Overview of testing conditions and practical recommendations for assessment of biofilm production by staphylococci. Apmis.

[47] Singh N.P., McCoy M.T., Tice R.R., Schneider E.L. (1988). A simple technique for quantitation of low levels of DNA damage in individual cells. Exp. Cell Res..

[48] Sardas S., Omurtag G.Z., Tozan A., Gül H., Beyoglu D. (2010). Evaluation of DNA damage in construction-site workers occupationally exposed to welding fumes and solvent-based paints in Turkey. Toxicol. Ind. Health.

